# The Reality of “Real-Life” Neuroscience: A Commentary on Shamay-Tsoory and Mendelsohn (2019)

**DOI:** 10.1177/1745691620917354

**Published:** 2020-04-21

**Authors:** Gijs A. Holleman, Ignace T. C. Hooge, Chantal Kemner, Roy S. Hessels

**Affiliations:** 1 Experimental Psychology, Helmholtz Institute, Utrecht University; 2 Developmental Psychology, Utrecht University; 3 Brain Center, University Medical Center Utrecht

**Keywords:** ecological validity, generalizability, ecological approach, definitions

## Abstract

The main thrust of Shamay-Tsoory and Mendelsohn’s ecological approach is that “the use of real-life complex, dynamic, naturalistic stimuli provides a solid basis for understanding brain and behavior” (p. 851). Although we support the overall goal and objectives of Shamay-Tsoory and Mendelsohn’s approach to “real-life” neuroscience, their review refers to the terms “ecological validity” and “representative design” in a manner different from that originally introduced by Egon Brunswik. Our aim is to clarify Brunswik’s original definitions and briefly explain how these concepts pertain to the larger problem of generalizability, not just for history’s sake, but because we believe that a proper understanding of these concepts is important for researchers who want to understand human behavior and the brain in the context of everyday experience, and because Brunswik’s original ideas may contribute to Shamay-Tsoory and Mendelsohn’s ecological approach. Finally, we argue that the popular and often misused concept of “ecological validity” is ill-formed, lacks specificity, and may even undermine the development of theoretically sound and tractable research.

In “Real-Life Neuroscience: An Ecological Approach to Brain and Behavior Research,” Shamay-Tsoory and Mendelsohn (2019) argue that “the field of cognitive neuroscience may be hampered by the limited ecological validity that characterizes the bulk of paradigms” (p. 841). To counter this problem, Shamay-Tsoory and Mendelsohn advocate for researchers to strive for experimental paradigms that possess “high ecological validity” (p. 851). The goal of Shamay-Tsoory and Mendelsohn’s ecological approach to cognitive neuroscience is to understand human behavior and the brain within the context of everyday human experience. To achieve this goal, Shamay-Tsoory and Mendelsohn advocate for “the use of real-life complex, dynamic, naturalistic stimuli provides a solid basis for understanding brain and behavior” (p. 851). Specifically, given that humans are active, embodied, social agents who interact with each other in everyday situations, experimental paradigms used by researchers must be aimed at preserving these characteristics. Finally, Shamay-Tsoory and Mendelsohn argue that with the advent of new portable measuring devices (e.g., mobile electroencephalography [EEG], functional near-infrared spectroscopy [fNIRS], and wearable eye trackers), the investigation of brain and behavior in “real life” has become a methodologically feasible goal for cognitive neuroscientists.

Although we support the goals and objectives of Shamay-Tsoory and Mendelsohn’s ecological approach, the aim of this commentary is to clarify two important concepts of psychological theory, namely *ecological validity* and *representative design*. In Shamay-Tsoory and Mendelsohn’s review, the authors acknowledge Egon Brunswik as the originator of these two concepts (p. 843). However, Shamay-Tsoory and Mendelsohn do not provide a definition of these concepts (neither Brunswik’s definitions nor their own), and the ways in which Shamay-Tsoory and Mendelsohn use these concepts is different from how Brunswik originally introduced them. We think it is important to clarify Brunswik’s original definitions, not just for history’s sake, but primarily because Brunswik’s ideas may contribute to Shamay-Tsoory and Mendelsohn’s ecological approach to study brain and behavior in everyday life. In what follows, we explain how Brunswik defined the concept of ecological validity and how it is related to his methodological program of representative design. Finally, we discuss how Shamay-Tsoory and Mendelsohn’s ecological approach may benefit from Brunswik’s perspective on the problem of generalizability.

## Brunswik’s Forgotten Legacy: Ecological Validity and Representative Design

**Fig. 1.**
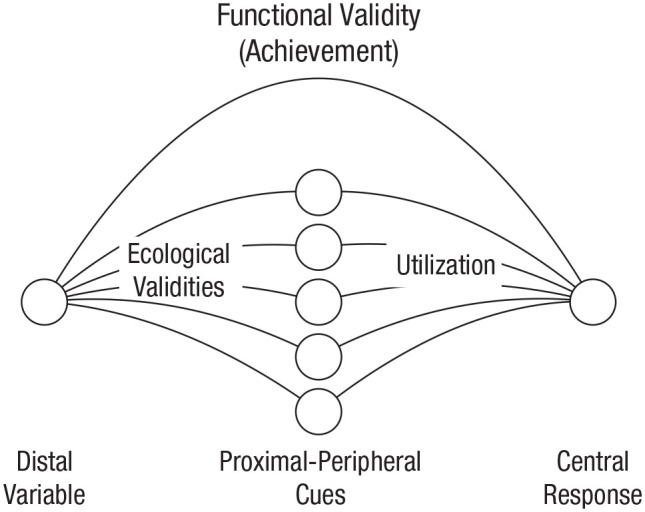
The lens model. Redrawn from Brunswik (1955, 1956b).

Brunswik (1952/1955, 1955, 1956b) defined ecological validity as the potential utility of a proximal cue (i.e., stimulus information) available to an organism to infer the state of a distal variable (e.g., object in the environment). To illustrate Brunswik’s definition of ecological validity, we take a situation in which a human observer judges the age of another person. Consider Brunswik’s lens model (Brunswik, 1955, 1956b), depicted in Figure 1. The distal variable in our example is the person-object’s age and the observer’s judgment is the central response. The degree to which the actual age of the person matches the observer’s judgment is what Brunswik called “functional validity,” or “achievement.” A variety of proximal cues (i.e., information sources) may be available and utilized by the observer to infer someone’s age. The correlation between the distal variable (i.e., age) and a proximal cue is called “ecological validity,” and the correlation between cues and the observer’s judgment (i.e., central response) is called “cue utilization.”

In Brunswik’s lens model, ecological validity and cue utilization can—but do not necessarily have to—correspond to each other. If, say, an observer infers someone’s age from a person’s physical appearance, the observer may use cues such as hair color, skin condition, posture, and clothing. Some of these cues may have “limited” or “low” ecological validity but can still be useful in perception. According to Brunswik (1956b), the observer may use “relatively superficial and stereotyped cues of limited ecological validity, preferably a multitude of them” (p. 89). In other words, the observer may use a variety of cues, some of which are more reliable than others, and these cues may be used interchangeably. In contrast to how Brunswik defined ecological validity, Shamay-Tsoory and Mendelsohn’s usage of ecological validity is quite different. Although Shamay-Tsoory and Mendelsohn do not explicitly define ecological validity, they seem to employ the phrase “ecological validity” to evaluate and compare lab-based research with “real life.” This is evident from several phrases in their review. For example, Shamay-Tsoory and Mendelsohn (2019) write that “the field of cognitive neuroscience may be hampered by the *limited ecological validity* [emphasis added] that characterizes the bulk of paradigms and settings in the field” (p. 841), and they write that “real-life situations provide a natural context and allow dynamic movement and feedback. . . . [C]ollecting rich data from real-life experiments offers the opportunity to evaluate multiple variables across experiments possessing *high ecological validity* [emphasis added]” (p. 841). Yet Brunswik’s lens model allows for “limited” or “low” ecological validities, albeit only in the sense that ecological validity describes the potential utility of a proximal cue (i.e., stimulus information) available to an organism to infer the state of a distal variable (e.g., object in the environment).

Thus, Brunswik did not use the concept of ecological validity to evaluate whether experimental research (or stimuli, tasks, and conditions) resembles or approximates “real life” (as is done by e.g., Neisser, 1976, p. 33; Sonkusare, Breakspear, & Guo, 2019, p. 1), nor did Brunswik imply that “more” or “greater” ecological validity warrants generalizability to the “real world”—as implied by, for example, Matlin (1989, p. 8) and Smilek, Birmingham, Cameron, Bischof, and Kingstone (2006, p. 104). Rather, Brunswik’s concern was that “ecological validities” can be dramatically misrepresented in certain (lab-based) experiments (Brunswik, 1943, 1956b; see also Hammond & Stewart, 2001). As an example of this concern, Brunswik (1943) describes the experimental procedures used by researchers in his day to study learning behavior in rats. He wrote that these experiments contain “Situations in which food can be found always to the right and never to the left, or always behind a black door and never behind a white one.” (p. 261). Thus, the door color or direction in the experimental setting perfectly predicts food location. According to Brunswik, the variables studied in these experiments are often artificially “tied” or “untied” (Brunswik, 1955), which can result in either perfect correlations between variables (i.e., ecological validities of −1 or +1) or in uncorrelated variables (i.e., ecological validities of 0). However, in a “natural ecology” (i.e., not the construction of an experimenter), the ecological validities between variables are rather somewhere in between. Thus, according to Brunswik (1943), the all-or-nothing reward schedules in these experiments are “not representative of the structure of the environment” (p. 261). To solve the limitations of such experimental designs, Brunswik devised his method of “representative design” (Brunswik, 1955, 1956b):

To obviate the intrinsic shortcomings of the artificial, “systematic” designs . . . I have advocated that in psychological research not only individuals be representatively sampled from well-defined “populations” but also stimulus situations from well-defined natural-cultural “ecologies”; only by such representative design of experiments can the ecological generalizability of functional regularities of behavior and adaptation be ascertained. (1956a, p. 159)

Simply put, representative design means that researchers first need to define a reference class of stimuli, tasks, and situations to which they intend to generalize a result. By sampling from this predefined set of conditions, the set of conditions toward which the generalization is intended is part of the experimental design. According to Brunswik (1956b, p. 53), “generalizability of results concerning the . . . variables involved must remain limited unless at least the range, but better also the distribution . . . of each variable, has been made representative of a carefully defined set of conditions.”

## The Reality of “Real-Life” Neuroscience

Although Brunswik’s method of representative design has been discussed much more thoroughly elsewhere (Araujo, Davids, & Passos, 2007; Dhami, Hertwig, & Hoffrage, 2004; Hammond & Stewart, 2001), here we primarily want to clarify a crucial difference between Brunswik’s representative design (1955, 1956b) and Shamay-Tsoory and Mendelsohn’s ecological approach. Brunswik believed that to justify claims about generalizability, one must first specify a reference class, or set of conditions toward which the generalization is intended. Thus, following Brunswik’s approach, experimental arrangements should be based on a well-defined set of conditions—that is, a representative sample of stimuli, tasks, and situations (i.e., in terms of their number, distribution, range, ecological validities, and intercorrelations of variables) that are deemed relevant to the context of functional behavior one is interested in. On the other hand, Shamay-Tsoory and Mendelsohn’s ecological approach primarily suggests enhancement of the “ecological validity” of experimental paradigms by conducting “real-life experiments” (p. 11) that include “real-life situations” (p. 11) and “real-life experiences” (p. 11). However, these “real-life” categories patently lack specificity. Without a more well-defined set of conditions, the notion of “real life” invokes a potentially infinite range of stimuli, tasks, situations and conditions that are logically and methodologically unfeasible to be represented in any formal manner. As Hammond, a former student and propagator of Brunswik’s research philosophy noted,

The real trouble with introducing the terms “real world” or “real life” and the reason they should be abandoned is that they are simply low-grade escape mechanisms; their use makes it unnecessary to define the conditions toward which the generalization is intended. One need only assume (without evidence) that everyone knows what these terms entail. (Hammond & Stewart, 2001, pp. 7–8)

Shamay-Tsoory and Mendelsohn’s ecological approach certainly has merit. Their review points to several limitations of certain lab-based experiments, and the authors identify areas of research in which new technological advances may be particularly useful to overcome these limitations, such as the fields of social cognition and episodic memory. Indeed, there have been numerous theoretical and methodological developments that support Shamay-Tsoory and Mendelsohn’s claims about the importance of the social context and potential for social interaction on behavioral and neurobiological functioning (see also De Jaegher, Di Paolo, & Gallagher, 2010; Risko, Richardson, & Kingstone, 2016; Schilbach et al., 2013), as also highlighted in their review. However, we think that Shamay-Tsoory and Mendelsohn’s ecological approach may also benefit from Brunswik’s most crucial lesson, namely that researchers should first define the contexts of behaviors that they are interested in and then show how these are well represented in their experiments. Thus, instead of subsuming research on brain and behavior during social interactions under the broad category of “real social interactions” (Shamay-Tsoory & Mendelsohn, 2019, p. 11), emphasis should be on specific types of social interactions. For example, the interpersonal and behavioral dynamics of parent-child interactions (Reindl, Gerloff, Scharke, & Konrad, 2018) may differ fundamentally from interactions between romantic partners (Kinreich, Djalovski, Kraus, Louzoun, & Feldman, 2017) or from student–teacher interactions in a classroom (Dikker et al., 2017). Different types of social interactions may be representative of a person’s “natural ecology” (as Brunswik might have put it) to various degrees and involve a diverse repertoire of cognitive and behavioral adaptations and responses. Consequently, we believe that specifying the interpersonal dynamics and the behavioral tasks and activities of social interactions is key to understanding their cognitive and neurobiological underpinnings. In this manner, we may begin to uncover the context-specific and the context-generic aspects of brain and behavior during “social interaction” at large.

To conclude, we think that a full-fledged ecological approach to brain and behavior should not merely be a matter of employing more “ecologically valid” and “real life” stimuli and settings. The problem of such an approach is that it may foster misguided ideas about what ecological validity means and what is entailed by “real life” (for a history, see Hammond, 1998). Nowadays, the concept of ecological validity is widely used as a catchall phrase to make intuitive claims about whether a given experiment approximates “real life” or whether one’s findings generalize to the “real world” (as previously pointed out by Hammond, 1998; Hammond & Stewart, 2001). Moreover, the concept of ecological validity is sometimes used as a blunt weapon to criticize experiments, typically in the absence of a proper definition or specific set of criteria (for further discussion, see Dunlosky, Bottiroli, & Hartwig, 2009; Schmuckler, 2001). Therefore, to more adequately evaluate and discuss whether a given experimental arrangement has captured the relevant aspects of cognitive and behavioral functioning within a particular context, it would be more constructive if researchers stopped using “ecological validity” as an easy substitute for “real life.” Instead, researchers need to be more explicit about their specific focus of inquiry and explain how and why certain experimental designs may or may not have captured the relevant characteristics of cognition and behavior that they are interested in. As Brunswik reminds us,

Mostly there is little technical basis for telling whether a given experiment is an ecological normal, located in the midst of a crowd of natural instances, or whether it is more like a bearded lady at the fringes of reality, or perhaps like a mere homunculus of the laboratory out in the blank. (1955, p. 204)

## Transparency

*Action Editor:* Aina Puce

*Editor:* Laura A. King
